# Development of a fuzzy logic-controlled system for home cultivation of sweet basil

**DOI:** 10.3389/fpls.2022.999106

**Published:** 2022-10-19

**Authors:** Jung-Sun Gloria Kim, Wooyoung Jeong, Seongmin Park, Myongkyoon Yang

**Affiliations:** ^1^ Department of Biosystems Engineering, Seoul National University, Seoul, South Korea; ^2^ Global Smart Farm Convergence Major, Seoul National University, Seoul, South Korea; ^3^ Department of Technology Management, Hanyang University, Seoul, South Korea; ^4^ Smart Agriculture Innovation Center, Kyungpook National University, Daegu, South Korea

**Keywords:** fuzzy logic, indoor farming, controlled environment agriculture, sweet basil, light quality, growth stage, home food gardening

## Abstract

As environmental pollution and the global population increase, and the COVID-19 pandemic becomes more severe, demands for indoor farming, especially home food gardening, have also increased. However, most research thus far has focused on large-scale food production, with very few studies having been conducted at the household scale. Also, the devices cultivating household crops with control systems in a continuous way, which minimize fluctuations of environmental conditions, have been rarely developed. Therefore, this study aimed to design a household cultivation system for sweet basil that is automatically and continuously controlled by fuzzy logic with a Raspberry Pi4. Three inputs (temperature, humidity, and growth stage) and seven outputs (fan, humidifier, heater 1, heater 2, LED red, green, and blue) were used with six rules, ensuring that three lights operated independently upon three growth stages. Simulation and actual operation were carried out, resulting in an appropriately controlled system that operated with few defects. In the case of an operation of the input variable, temperature and humidity were maintained at an average of 21.24 °C and 75.58%, respectively, and the LED operation for the growth stage was confirmed to be flawless. For verification of the designed fuzzy system, a comparison between the simulation and actual operation was performed to examine differences and identify problems. To this end, Pearson’s correlation coefficients were used, and the direction of correction of the fuzzy logic system was proposed. Through these results, the feasibility of a home cultivation system using fuzzy logic was demonstrated, and it is expected that further studies applying it will be conducted in the future.

## Introduction

According to the United Nations, the global population is expected to reach 9.7 billion by 2050, rising continuously from 7.9 billion in January 2022 (Duro et al., 2020). Thus, potential food shortages should be mitigated by increasing food production to meet future demands from a growing population. To add to this issue, environmental pollution is also worsening. In this context, farming techniques, such as indoor farming, that require less land and decreased resistance to adverse climatic conditions, are potentially important solutions (Stein, 2021; Mitchell, 2022). In terms of controlled environment agriculture (CEA), indoor farming includes various methods such as vertical farms, zfarms, and greenhouses, all of which involve environmental conditions that are controlled for optimal cultivation.

Lately, household food gardening has become increasingly popular due to the COVID-19 pandemic (Mullins et al., 2021). In 2020, 18.3 million Americans participated in gardening for the first time, 49% of whom started because of the pandemic. Additionally, a survey in Canada showed that 51% of respondents grew at least one type of fruit or vegetable at home and 17.4% of those started home food gardening during the pandemic (Mullins et al., 2021). Despite the increasing importance of home food gardening, only a handful of studies have been conducted on this topic.

Within the fundamental aspects that are considered in agriculture and that should also be considered in domestic agriculture, are the phenological stages of growth. The BBCH growth stage scale is a framework used to judge plant growth and to define the phenological growth stages of various crops (Boyes et al., 2001). Accordingly, several authors have introduced this framework to various crop species (Meier et al., 2009; Adiga et al., 2019; Enriquez-Hidalgo et al., 2020).

Plant growth is modulated by light, which affects seed germination, transition to flowering, and adaptation to stressors. Plants can sense and respond to a wide range of spectra, spanning from UV-C (260 nm) to the far-red (720–780 nm) region. Combinations of different light wavelengths affect plant growth, metabolism, and morphology. Many studies have confirmed the effects of light quality on plants, for example, some light treatments increase the volatile content, mass, and antioxidant capacity in basil. Additionally, narrow-bandwidth illumination can induce discrete changes in volatile composition that affect sensory quality in commercial herbs (Carvalho et al., 2016). Furthermore, studies show that the appropriate use of LEDs improves production efficiency *via* phytochemical enrichment (Taulavuori et al., 2017). Recently, a novel light combination was proposed (Rihan et al., 2020). Generally, 450 nm blue light is used commercially, but two absorbance peaks were observed in the photosynthetically active radiation region at 435 nm and 665 nm. Therefore, a ratio of 1.4 blue to 1.0 red increased growth and yield compared to a 1:1 blue: red ratio. Despite the apparent importance of light for plant growth, very few studies have tried to adjust light quality according to different plant growth stages.

To control actuators and enhance the effectiveness of the operation, the system needs to be operated by some logic. Fuzzy logic has been used in various appliances such as electric rice cookers and air conditioners because it is free from the binary approach (i.e., it records the degree of validity) (Badami and Stephanou, 1997). Unlike fuzzy logic, crisp logic is the same as Boolean logic (i.e., either 0 or 1). By contrast, fuzzy logic is continuously valued, has membership functions, and considers the degree of membership. Fuzzy logic is highly effective in feedback control systems and is generally easy to implement. The computational structure of fuzzy logic is composed of fuzzification, inference engines, and defuzzification modules. For example, fuzzification translates the numerical values for temperature errors into linguistic value such as “low,” “very low,” “zero,” “high,” or “very high.” A fuzzy inference engine infers fuzzy outputs using fuzzy implications and the rules of inference of fuzzy logic. Defuzzification uses the fuzzy output of the rules to generate an output formulated as “crisp” numeric values, which are used as the control input. The relationship between input and output is in the form of “If-Then” rules that are based on the dynamic performance of processes (Potdar et al., 2017). Fuzzy logic has been used to manage environmental conditions in a range of agricultural circumstances, such as general crop- (Singhala et al., 2014; Robles Algarín et al., 2017; Xu et al., 2017; Lakhiar et al., 2018), pest- (Roussel et al., 2000; Shi et al., 2007; Jesus et al., 2008), and irrigation management (Dengel, 2013; Bannerjee et al., 2018; Daud et al., 2018; Jayalakshmi et al., 2020). However, no studies have reported applying fuzzy logic to manage light quality according to different plant growth stages. The closest example used the input variable “light” with three membership functions of “light low,” “light optimal,” and “light high” (Iliev et al., 2014).

A representative problem of indoor farming is the high cost of the initial setup for cultivating inexpensive crops, such as lettuce. Therefore, the discovery and improvement of competitive crops are essential. Sweet basil (*Ocimum basilicum* L.) could be such a species, according to multiple studies (Carvalho et al., 2016; Liaros et al., 2016). The demand for this herb is increasing with consumers’ awareness of its unique aroma and taste, but arguably this demand is not yet at its peak. For this reason, this study aimed to set up a system to cultivate sweet basil.

Therefore, the goal of this study was to develop an automatically controlled home cultivation system using fuzzy logic so that it proves actuators operate appropriately according to sensors based on the designed logic in the systematic aspect. Several input and output variables were utilized, including growth stage and light quality, and several fuzzy system elements were designed for this purpose. In many existing studies using fuzzy system, there was no proposed modification method to examine the vulnerable components of a given fuzzy logic design. Furthermore, most studies only simulated systems and did not actually implement them. That’s why it has been hard to find out if the system can be actually operated as intentioned in reality. Hence, this study aimed to: (1) design an automatic cultivation system targeting a specific species suitable for home food production; (2) control a system using fuzzy logic considering light quality associated with plant growth stage; (3) compare the simulation results to an actual operation; and (4) assess fuzzy logic using Pearson’s correlation coefficient to find out the weakness of the designed fuzzy logic.

## Materials and methods

### Target crop

As mentioned in the introduction, the target crop in this study is sweet basil. The conditions for cultivating sweet basil in an environment controlled by a fuzzy logic system were selected considering its market value. According to the herb gardening guide from the University of Illinois Extension website, sweet basil is sensitive to cold temperatures and therefore its media temperature should be at least 21.1 °C (Illinois, 2022). Furthermore, basil growth becomes stunted under 10 °C, with damaged and blackened leaves. In terms of humidity, most plants require 45–75%, but basil requires 60–65% and it rapidly perishes in dry airflow (Luv2garden, 2020). The fuzzy logic control system was designed based on these environmental preferences.

### Designing the cultivator

**Figure 1:**
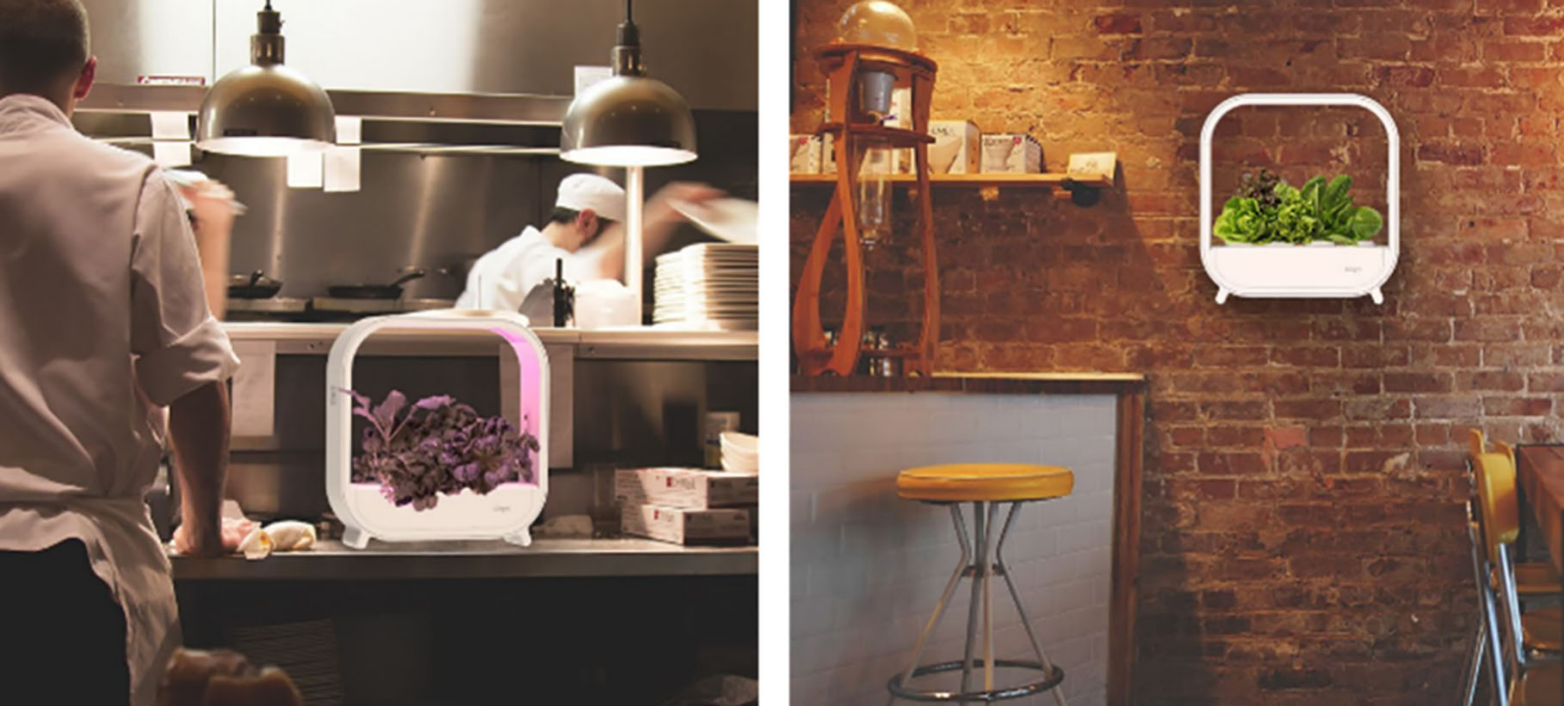
Concept design for the two modes of operation: standing (left), and wall-mounted (right).

The cultivator was designed to be employed in two ways in order to use of space more efficiently: standing and wall-mounted (**Figure 1**). **Figure 1** shows concept images of the two ways in a cultivator, virtually showing its expecting uses. The product was prototyped using acrylonitrile butadiene styrene (ABS) material. The structure of the cultivator was partially closed but not completely sealed, with two detachable transparent acrylic covers attached by 12 magnets. Six magnets were set on the front and six were set on the back, where two holes were made in the edges in order to hang the cultivator on a wall. Four pots were placed in the cultivator, which is a reasonable quantity for growing herbs at home.

**Figure 2:**
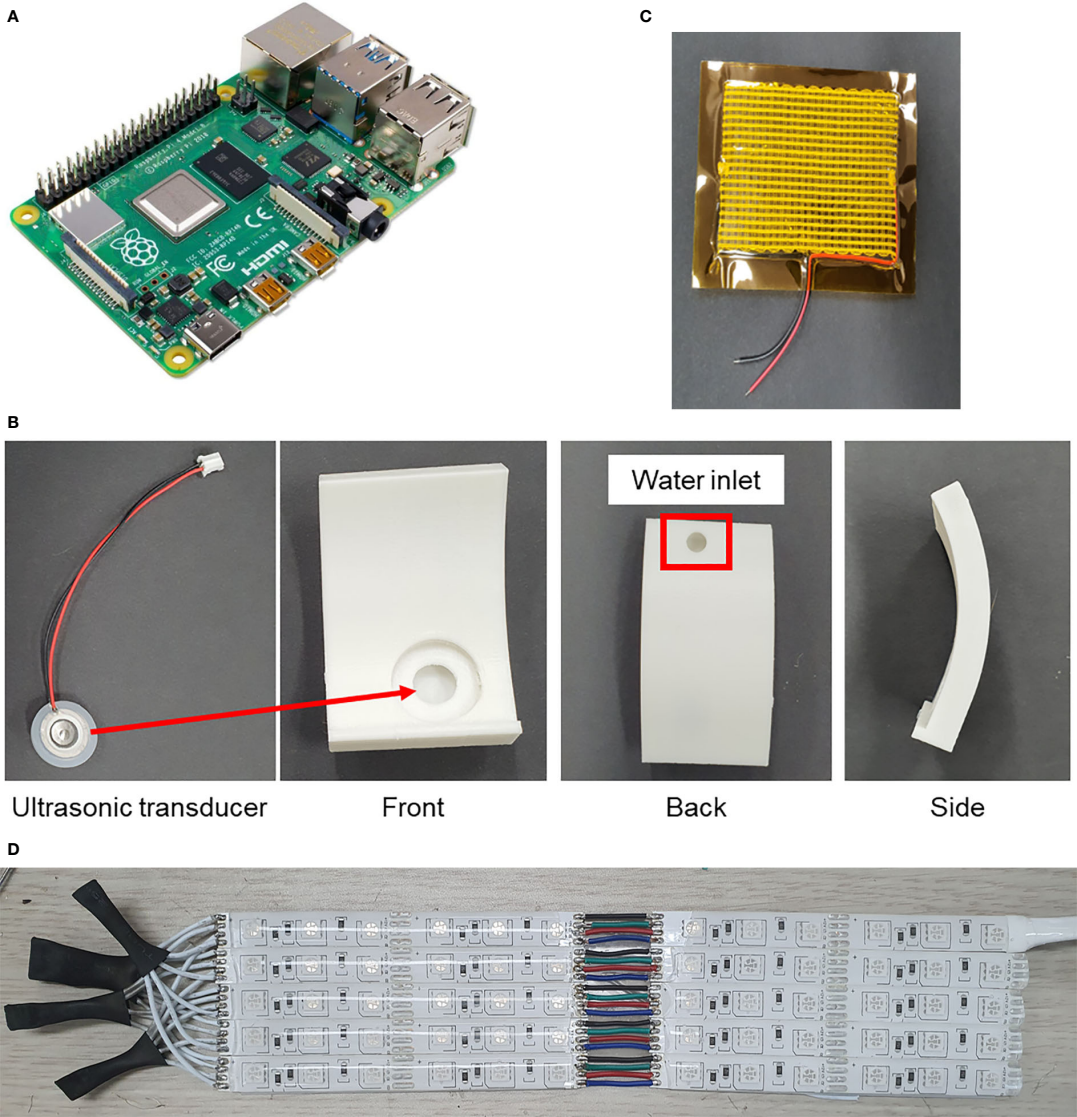
Main elements of the cultivator. **(A)** Raspberry Pi 4, **(B)** water tank and ultrasonic humidifier, **(C)** heating wire pad, and **(D)** tailored RGB LEDs.

**Figure 3:**
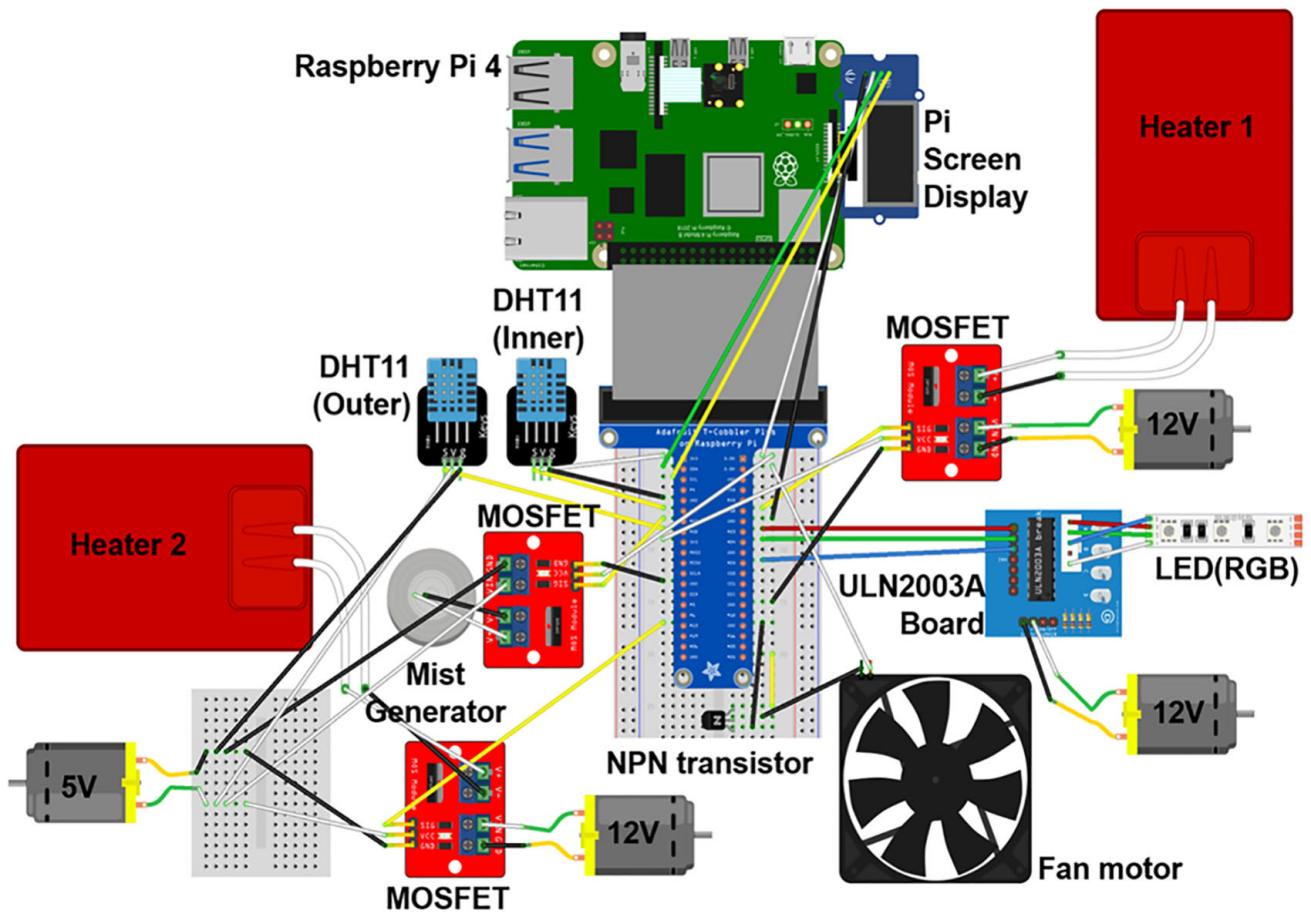
Circuit design between Raspberry Pi and the other components.

**Figure 2** shows the main components used in this study. A Raspberry Pi (Raspberry Pi 4 model B, Raspberry Pi Foundation, UK) was selected to operate and control the system since nearly real-time data can be saved from multiple sensors and it enables additional complicated applications in the long term. A DHT11 sensor was used to measure temperature and humidity, while two heaters were used to control the temperature. A humidifier was 3D printed and inserted into the cultivator, and an ultrasonic transducer was attached to it. The humidifier was used to control both temperature and humidity. A fan was added for ventilation and to decrease the temperature in combination with the humidifier. In addition, RGB LEDs were tailored to independently control the light quality. For analog control of the system, a ULN2003 stepper motor driver board was used for the LEDs, an IRF520 MOSFET driver module for the heaters, a Grove water atomization module for the humidifier, and an NPN transistor (C3198) for the fan. The connection between the Raspberry Pi and the other components is shown in **Figure 3**. In terms of power supply, three 12 V AC/DC adapters were added for the heaters, as well as one 5 V SMPS for the humidifier, DHT11, and the MOSFET module, connected in parallel.

**Figure 4:**
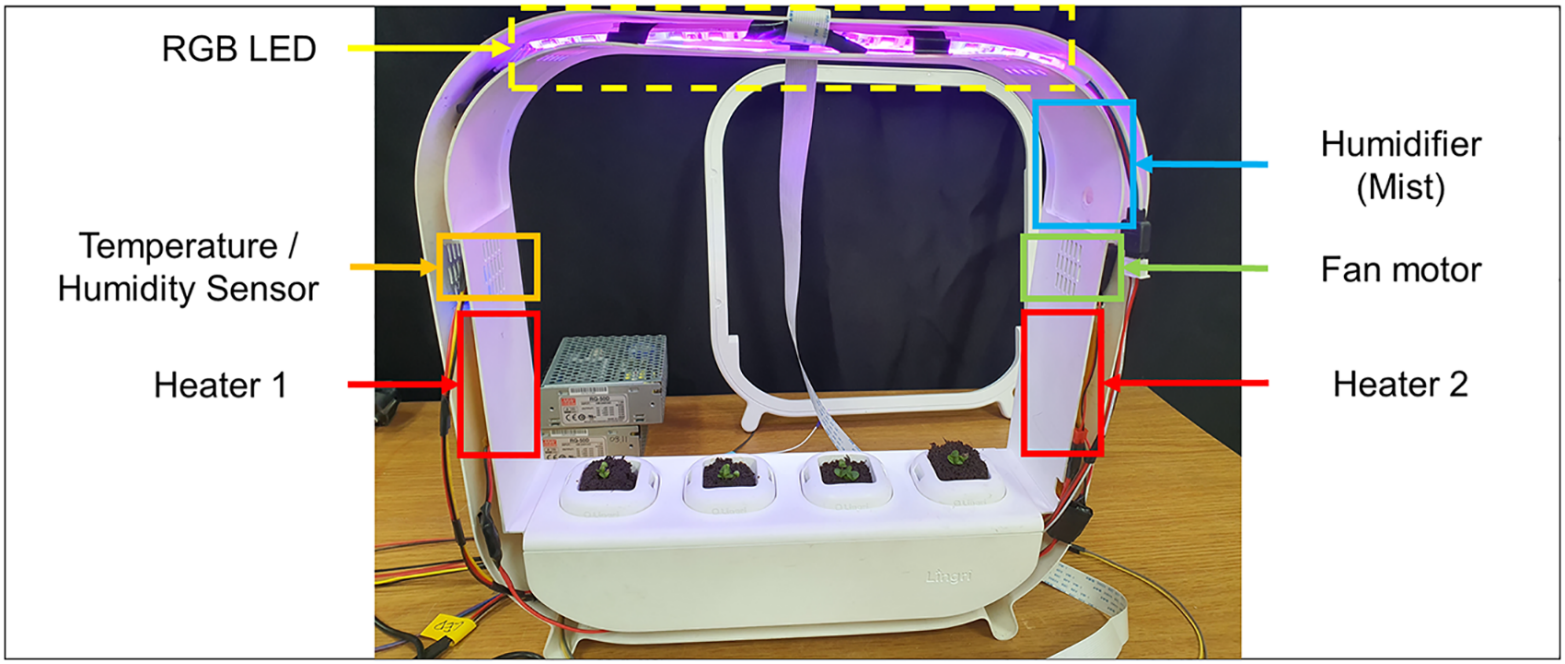
Composition of the main components in the cultivator.

The fabricated system is shown in **Figure 4**. RGB LEDs were placed on top to provide light to the plants, a humidifier was placed on the upper side of the right pillar, and a fan motor was installed below the humidifier to spread the mist. To simplify the variable name, mist and humidifier were used interchangeably. A temperature and humidity sensor was placed on the upper side of the left pillar, at a distance from the mist. The heaters were attached to the lower side of each pillar to deliver heat evenly.

### Fuzzy logic system

**Figure 5:**
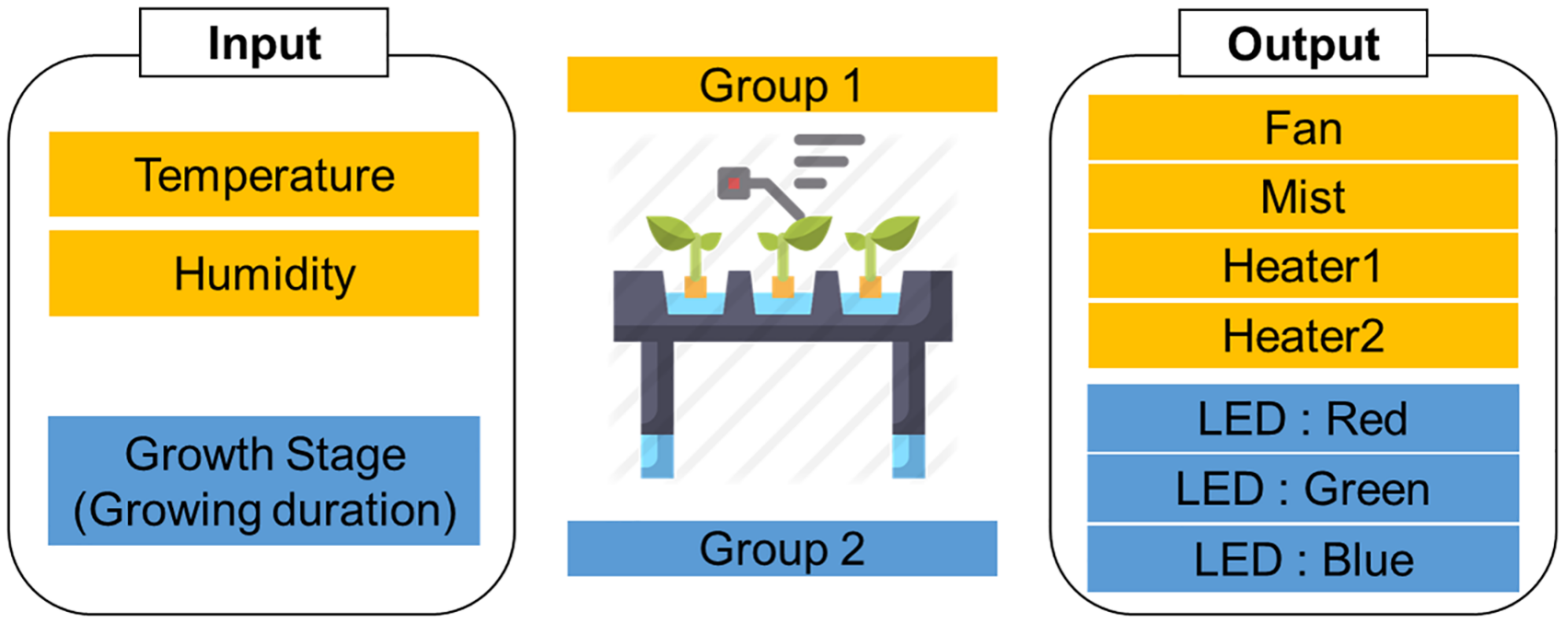
Schematic design of the input and output variables.

A fuzzy logic system was used for analog control of each environmental condition. The system was designed from the systematical point of view to confirm the robust actual operation as programmed considering biological elements such as plant growth levels. The existing studies regarding to control environments of indoor farming have the limitations of not considering biological elements. This study considered the impact of lighting on plants when designing this system. Since this is a rare try in studies, it did not jump to prove difference of crop yields at once. Firstly, temperature and humidity are important environmental elements and they can be controlled by a fan, a mist, a heater. Secondly, this study selected the time duration as plant growth levels which means numerical inputs instead of images representing morphological characteristics. Red, blue, and green LEDs should be independently modulated upon plant growth levels. For these reasons, the system consisted of three inputs and seven outputs, as shown in **Figure 5**. The input variables were temperature, humidity, and growth stage and the output variables were the fan, mist, heater 1, heater 2, red LED, green LED, and blue LED. These variables were divided into two groups. Group 1 included temperature and humidity, as well as the related outputs, fan, mist, heater 1, and heater 2. Group 2 included the growth stage and its related outputs red LED, green LED, and blue LED. The novel point is that three different colored LEDs were independently assigned as output variables to control light quality depending on the growth stage.

To perform fuzzification, the three input variables (temperature, humidity, and growth stage) were set. The growth stages of many crops have been studied according to the BBCH scale; however, that is not the case for sweet basil. In this study, the plant growth level was defined until harvest, which was considered to be the edible stage. Therefore, growth was partitioned into three stages according to phenotypic characteristics, which correspond to numeric codes on the BBCH scale: 09 (cotyledons or coleoptile break through the soil surface), 10 (first true leaf emerges from coleoptile; or cotyledons completely unfold), and 51 (inflorescence or flower buds become visible). These were used as the three growth stages of germination, vegetative growth, and flowering, respectively.

**Figure 6:**
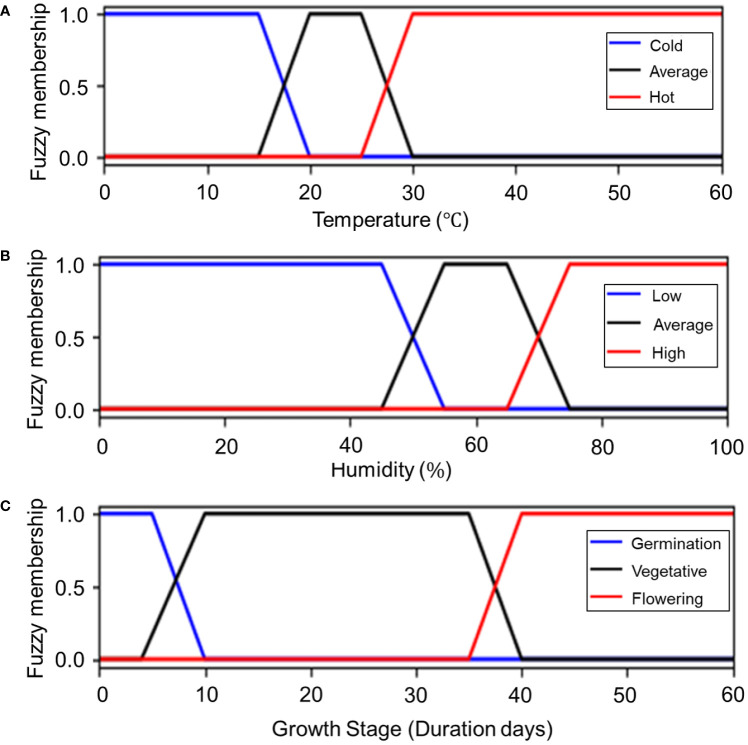
Membership functions of input variables. **(A)** Temperature, **(B)** humidity, and **(C)** growth stage.

The membership functions (μ) for the input variables were as follows. The name of the member function had the structure [u variable level]. As all the membership functions were designed based on basil cultivation requirements, the system identified an average temperature of 20–25 °C and 55–65% humidity. Furthermore, because germination periods vary between seeds, the germination growth stage was set at five to seven days. Although the flowering stage depends on crop management, it generally begins on the 35^th^ day, wherein the vegetative stage comes to an end. Accordingly, each input variable had three levels so that nine formulas could be obtained. The combined graph for the three input variables is shown in **Figure 6**.


(1) $$\mu TemperatureCold\left(x\right)=\{\begin{array}{c} 1\;\;\;\;\;\;\;x<15\\ \frac{20-x}{5}15\leq x<20\\ 0\;20\leq x \end{array}$$

(2) $$\mu TemperatureAverage\left(x\right)=\{\begin{array}{c} 0x<15\;or\;30\leq x\\ \frac{x-15}{5}\;\;\;\;\;\;15\leq x<20\\ 1\;\;\;\;\;\;\;20\leq x<25\\ \frac{30-x}{5}\;\;\;\;\;\;25\leq x<30 \end{array}$$

(3) $$\mu TemperatureHot\left(x\right)=\{\begin{array}{c} 0\;\;\;\;\;\;\;\;\;\;x<25\\ \frac{x-25}{5}\;\;\;25\leq x<30\\ 1\;\;\;\;30\leq x \end{array}$$

(4) $$\mu HumidityLow\left(y\right)=\{\begin{array}{c} 1\;\;\;\;\;\;\;\;\;\;\;y<15\\ \frac{55-y}{10}\;\;\;\;45\leq y<55\\ 0\;\;\;\;\;55\leq y \end{array}$$

(5) $$\mu HumidityAverage\left(y\right)=\{\begin{array}{c} 0\;\;\;\;\;\;y<45\;or\;75\leq y\\ \frac{y-45}{10}\;\;\;\;\;45\leq y<55\\ 1\;\;\;\;\;\;55\leq y<65\\ \frac{75-y}{10}\;\;\;\;\;65\leq y<75 \end{array}$$

(6) $$\mu HumidityHigh\left(y\right)=\{\begin{array}{c} 0\;\;\;\;\;\;\;\;\;\;\;y<65\\ \frac{y-65}{10}\;\;\;\;65\leq y<75\\ 1\;\;\;\;\;75\leq y \end{array}$$

(7) $$\mu GrowthStageGermination\left(z\right)=\{\begin{array}{c} 1\;\;\;\;\;\;\;\;\;\;\;z<5\\ \frac{10-z}{5}\;\;\;\;\;5\leq z<10\\ 0\;\;\;\;\;10\leq z \end{array}$$

(8) $$\mu GrowthStageVegetative\left(z\right)=\{\begin{array}{c} 0\;\;\;\;\;z<4\;or\;40\leq z\\ \frac{z-4}{6}\;\;\;\;4\leq z<10\\ 1\;\;\;\;10\leq z<35\\ \frac{40-z}{5}\;\;\;35\leq z<40 \end{array}$$

(9) $$\mu GrowthStageFlowering\left(z\right)=\{\begin{array}{c} 0\;\;\;\;\;\;\;\;\;\;\;z<35\\ \frac{z-35}{5}\;\;\;\;35\leq z<40\\ 1\;\;\;\;\;40\leq z \end{array}$$

In order to control the degree of operation of the device, pulse width modulation (PWM) was used to produce a continuous output with digital means. PWM uses a square pulse wave whose pulse width is modulated, leading to variations in the average value of the waveform. The average value of the signal $\bar{y}$ ) directly depends on the duty cycle, which is the proportion of “HIGH” time to regular interval or “period” of time and is expressed as a percentage of “on” time. A lower duty cycle yields lower power so that the power stays “off” for most of the time. As all of the output variables were used in combination with continuous outputs, the x-axes ranged from zero to 100% on the basis of duty cycles. The average value of the signal is as follows:


$$\bar{y}\;\;=\;\frac{1}{T}\int_{0}^{T}f\left(t\right)dt\;$$

$$\bar{y}\;\;=\;\frac{1}{T}\;\left(\int_{0}^{DT}y_{max}dt+\;\int_{DT}^{T}y_{min}dt\;\right)$$

$$\;\;\;\;=\;\frac{1}{T}\;\left(D\cdot T\cdot y_{max}\;+\;T\left(1-D\right)y_{min}\right)$$

(10) $$\;\;\;\;=D\cdot y_{max}+\left(1-D\right)y_{min}$$

Where *f*(*t*) is the waveform with period *T*, *y_min_* is the low value, *y_max_* is the high value, and *D* is the duty cycle.

Regarding LEDs, several factors had be considered when setting the three light regimes, including conflicting reports on their effects. For example, Aldarkazali et al. (2019) asserted that green light interferes with the growth and development of basil and that BR LEDs have a significant positive effect on the quantity and quality of essential oils that can be extracted from this crop. On the other hand, Carvalho et al. (2016) stated that RGB lighting and BR lighting do not affect the basil phenotype. In this study, green light was not excluded because it can penetrate the plant canopy, meaning that it helps plant growth under foliage or within proximity to other plants. Blue was expected to remain in a higher position except during the flowering stage, which was dominated by red and green light.

**Figure 7:**
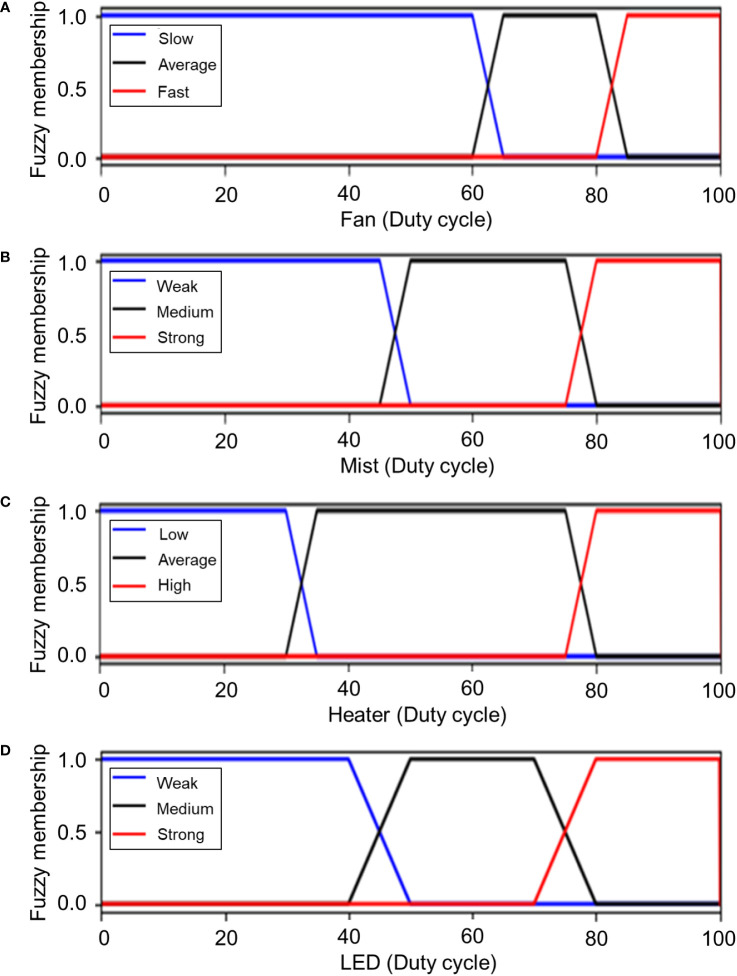
Membership functions of output variables. **(A)** Fan, **(B)** humidifier, **(C)** heater, and **(D)** LEDs.


The name of each member function for the output variable was the same as in the input method. For the seven output variables, four variables were defined by grouping each of the heater (heater 1, heater 2) and LED (red, green, blue) variables, which have overlapping member functions. All of the output variables were configured in three steps to obtain 12 formulas as in the following equations; the combined graph is shown in **Figure 7**.

(11) $$\mu FanSlow\left(i\right)=\{\begin{array}{c} 1\;\;\;\;\;\;\;\;\;\;\;i<60\\ \frac{65-i}{5}\;\;\;\;60\leq i<65\\ 0\;\;\;\;\;65\leq i \end{array}$$

(12) $$\mu FanAverage\left(i\right)=\{\begin{array}{c} 0\;\;\;\;\;i<60\;or\;85\leq i\\ \frac{i-60}{5}\;\;\;\;\;60\leq i<65\\ 1\;\;\;\;\;\;65\leq i<80\\ \frac{85-i}{5}\;\;\;\;\;80\leq i<85 \end{array}$$

(13) $$\mu FanFast\left(i\right)=\{\begin{array}{c} 0\;\;\;\;\;\;\;\;\;\;\;\;i<80\\ \frac{i-80}{5}\;\;\;\;\;80\leq i<85\\ 1\;\;\;\;\;\;85\leq i \end{array}$$

(14) $$\mu MistWeak\left(j\right)=\{\begin{array}{c} 1\;\;\;\;\;\;\;\;\;\;\;\;j<45\\ \frac{50-j}{5}\;\;\;\;\;45\leq j<50\\ 0\;\;\;\;\;\;50\leq j \end{array}$$

(15) $$\mu MistMedium\left(j\right)=\{\begin{array}{c} 0\;\;\;\;\;j<45\;or\;80\leq j\\ \frac{j-45}{5}\;\;\;\;\;45\leq j<50\\ 1\;\;\;\;\;\;50\leq j<75\\ \frac{80-j}{5}\;\;\;\;\;75\leq j<80 \end{array}$$

(16) $$\mu MistStrong\left(j\right)=\{\begin{array}{c} 0\;\;\;\;\;\;\;\;\;\;\;\;j<75\\ \frac{j-75}{5}\;\;\;\;\;75\leq j<80\\ 1\;\;\;\;\;\;80\leq j \end{array}$$

(17) $$\mu HeaterLow\left(k\right)=\{\begin{array}{c} 1\;\;\;\;\;\;\;\;\;\;\;\;k<30\\ \frac{35-k}{5}\;\;\;\;\;30\leq k<35\\ 0\;\;\;\;\;\;35\leq k \end{array}$$

(18) $$\mu HeaterAverage\left(k\right)=\{\begin{array}{c} 0\;\;\;\;\;\;k<30\;or\;80\leq k\\ \frac{k-30}{5}\;\;\;\;\;\;30\leq k<35\\ 1\;\;\;\;\;\;\;35\leq k<75\\ \frac{80-k}{5}\;\;\;\;\;\;75\leq k<80 \end{array}$$

(19) $$\mu HeaterHigh\left(k\right)=\{\begin{array}{c} 0\;\;\;\;\;\;\;\;\;\;\;\;k<75\\ \frac{k-75}{5}\;\;\;\;\;75\leq k<80\\ 1\;\;\;\;\;\;80\leq k \end{array}$$

(20) $$\mu LEDWeak\left(l\right)=\{\begin{array}{c} 1\;\;\;\;\;\;\;\;\;\;\;\;l<40\\ \frac{50-l}{10}\;\;\;\;\;40\leq l<50\\ 0\;\;\;\;\;\;50\leq l \end{array}$$

(21) $$\mu LEDMedium\left(l\right)=\{\begin{array}{c} 0\;\;\;\;\;l<40\;or\;80\leq l\\ \frac{l-40}{10}\;\;\;\;\;40\leq l<50\\ 1\;\;\;\;\;\;50\leq l<70\\ \frac{80-l}{10}\;\;\;\;\;70\leq l<80 \end{array}$$

(22) $$\mu LEDStrong\left(l\right)=\{\begin{array}{c} 0\;\;\;\;\;\;\;\;\;\;\;\;l<70\\ \frac{l-70}{10}\;\;\;\;\;70\leq l<80\\ 1\;\;\;\;\;\;80\leq l \end{array}$$

After fuzzification, the fuzzy rules were applied as the basis of the controller to manage the output variables. The fuzzy rules were then divided into two groups. In Group 1, the fan and heater were configured to run quickly and powerfully to dissipate heat in cold or high humidity conditions. Simultaneously, the humidifier was activated to prevent it from drying out and becoming cold. When the temperature and humidity reached the average, all of the devices operated at the average speed.

Group 2, however, needed a rule for lighting. Blue light dominated the germination and vegetative phases. Because germinating seeds require a darker space, we set the blue light to average and the rest to low during this period. Vegetative growth requires higher levels of light so we adjusted the blue light to high and the rest to medium. During the flowering stage, the red light should be more intense and it was configured accordingly.

After the input variables had undergone fuzzification and rules-based inference, clipping of the membership functions of the output variables was carried out. Following this, the clipped results were aggregated to be defuzzied. The defuzzied values were calculated using the centroid technique as the crisp output for control. All of the fuzzy rules are shown in **Tables 1** and **2**.

**Table 1:** Fuzzy associative matrix (Group 1).

Temperature	Humidity		
	Low	Average	High
**Cold**	–	–	FanFast MistWeak HeaterHigh
**Average**	–	FanAverage MistMedium HeaterAverage	–
**Hot**	FanSlow MistStrong HeaterLow	–	–

**Table 2:** Fuzzy associative matrix (Group 2).

Growth stage	LED color		
	Red	Green	Blue
**Germination**	Weak	Weak	Medium
**Vegetative**	Medium	Medium	Strong
**Flowering**	Strong	Strong	Weak

Simulation and verification were performed to verify the operation of the system through hardware design and fuzzy logic. Temperature and humidity were repeatedly measured and recorded inside and outside the cultivator for 40 days. The cultivator was placed within an office similar to a home environment. For statistical analysis, inter-quartile range (IQR) was used to eliminate outliers. The simulation was implemented using the Fuzzy Logic Toolbox provided by MATLAB (R2020b, MATLAB 9.9, MathWorks), and the actual operation was conducted with Python 3.6 in consideration of its commercial feasibility.

## Results and discussion

### System simulation

**Figure 8:**
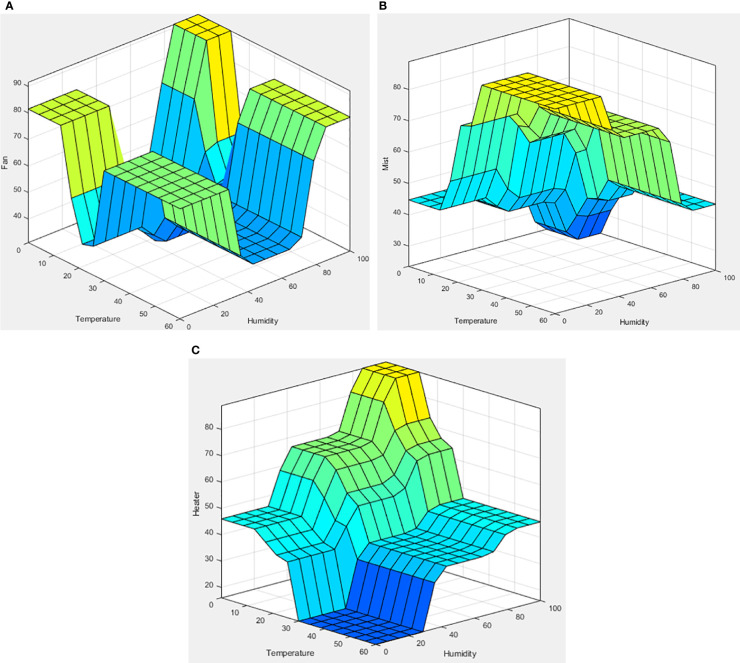
Surface view of the fuzzy logic simulation. **(A)** Fan, **(B)** humidifier, and **(C)** heaters (heater 1 and heater 2).

**Figure 9:**
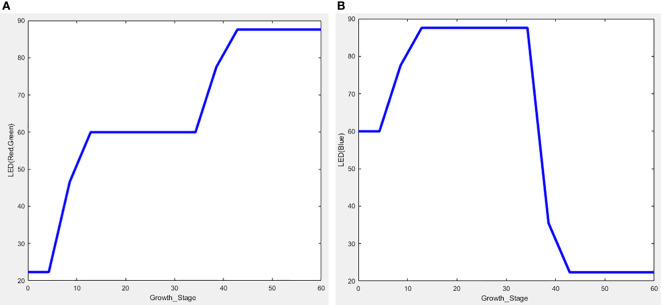
Surface view of the fuzzy logic simulation. **(A)** LED (red, green), **(B)** LED (blue).

The Fuzzy Logic Toolbox shows the simulation results in a graphic called “surface view” using the input fuzzy membership function and rules (**Figure 8**). The fan was designed to operate on its lowest setting when the temperature and humidity reached the average. The cross on the surface view indicates that the fan worked as intended. The mist was supposed to operate less when the temperature was low or the humidity high, and operate more when the temperature was high or the humidity low. The surface view shows that this was the case. The heaters were set to the opposite rules than those of the mist, and the simulation results confirm they operated as expected. Since the LED was the result of one input (the growth stage), a two-dimensional view was generated (**Figure 9**). Thus, the intended output of red, green, and blue appeared according to the growth stage.

### Operation analysis

#### Verification of system behavior

The temperature and humidity results are shown in **Table 3**. The outer temperature exhibited greater variations compared to the inner temperature. Within the cultivator the temperature ranged between 16 and 31 °C, with an average of 21.2 °C, while the external temperature ranged from 12 to 31 °C with a mean value of 19.8 °C. Ideally, basil should be grown between 21 and 26 °C, and certainly not below 18 °C. Therefore, basil should have a survival rate > 75% within this cultivator, given that the first and third quartiles were 18 and 25 °C, respectively. By contrast, basil would have a survival rate < 75% outside the cultivator because the first, second, and third quartiles were 16, 18, and 23 °C, respectively. This demonstrates that the environment within the cultivator was more stable than that outside it.

**Table 3:** Statistical analysis of temperature and humidity.

	InnerTemperature (°C)	InnerHumidity (%)	OuterTemperature (°C)	OuterHumidity (%)
**Mean**	21.24	75.58	19.81	16.57
**Std**	3.71	10.28	4.70	7.30
**Min**	16.00	49.00	12.00	5.00
**25%**	18.00	69.00	16.00	10.00
**50%**	20.00	75.00	18.00	15.00
**75%**	25.00	82.00	23.00	22.00
**Max**	31.00	95.00	31.00	39.00

**Figure 10:**
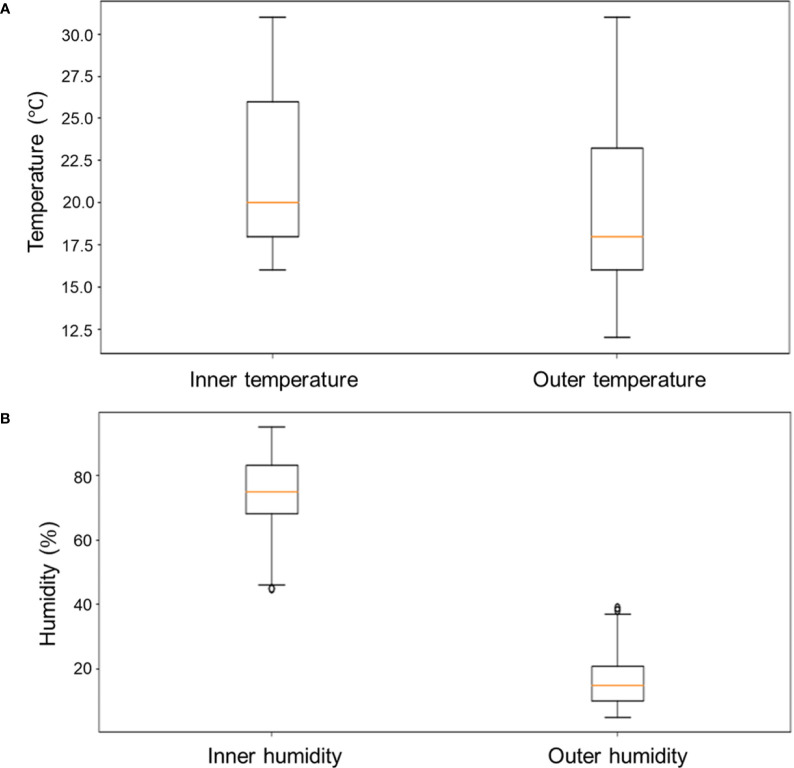
Comparison of boxplots according to the internal and external conditions of the cultivator. **(A)** Temperature and **(B)** humidity.

The outer humidity level was substantially lower than that within the cultivator. The difference in humidity inside and outside was much more pronounced than that in temperature (**Figure 10**). Research shows that basil withers rapidly in dry conditions (Luv2garden, 2020) and the optimum humidity for basil is between 60% and 80%, which is higher than that required for many other plants (Mijani et al., 2013; Pennisi et al., 2019; Song et al., 2020). The outer humidity was affected by human activities and its changes were unpredictable. The inner humidity measurements showed a pattern suggesting the humidifier worked as intended; when the humidity was reduced, the humidifier was switched on in order to increase it. Therefore, the conditions within the cultivator were more conducive to basil growth than those outside it as the mean inner and outer humidity levels were 75.58% and 16.57% respectively. The differences between the outer and inner temperature and humidity are presented in **Figure 10**.

Since the growth stage is a fixed value, the operation of the LEDs was checked to determine proper functioning. **Table 4** shows the values of each LED duty cycle automatically controlled by the fuzzy algorithm according to the growth stage up to the 40^th^ day. Following the initial germination period, (Days 5 to 10) as the growth stage changed to vegetative growth, a change was also confirmed in each LED value. The duty cycle of red and green increased from 22.5% to 60%, and the duty cycle of blue increased from 60% to 87.36%. This confirmed that the blue LED value was greater than that of the other LEDs, in accordance with the fuzzy rule condition.

**Table 4:** LED duty cycle change for duration of the study.

Duration (days)	LED color		
	Red	Green	Blue
**1**	22.59	22.59	60.00
**5**	26.75	26.75	63.08
**6**	31.66	31.66	66.43
**7**	37.06	37.06	70.23
**8**	43.22	43.22	74.70
**9**	50.64	50.64	80.25
**10**	60.00	60.00	87.36
**36**	64.17	64.17	66.96
**37**	68.75	68.75	52.62
**38**	73.94	73.94	41.31
**39**	80.01	80.01	31.57
**40**	87.36	87.36	22.59

The duty cycle of each LED converged to a similar level until the 35^th^ day (i.e., the flowering period) and was automatically controlled until the 40^th^ day, and then maintained. From the beginning of vegetative growth, the red and green duty cycle increased from 60% to 87.36% and the blue duty cycle decreased from 87.36% to 22.59%, confirming that the fuzzy control worked well. The duty cycle of red and green light was lower than that of blue at a ratio of 2.66 for seedlings and 1.46 for asexual growth, as intended. Furthermore, the ratio of red and green at flowering was 3.87, which was higher than that of blue.

#### Comparison between simulation and actual operation

**Figure 11:**
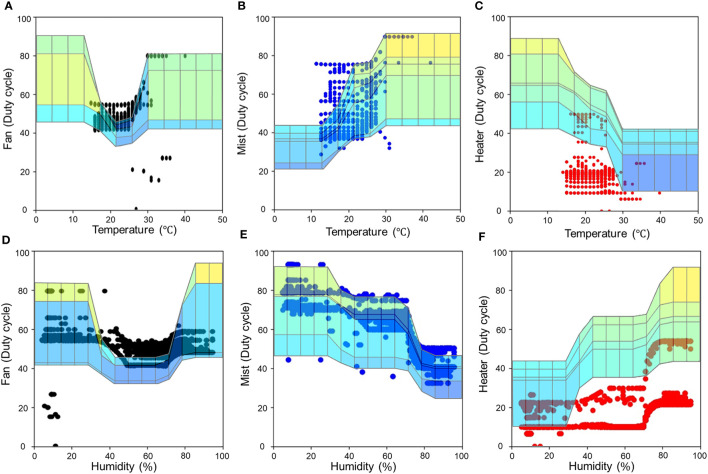
Comparison of simulated and actual operational results. **(A)** Temperature-fan, **(B)** temperature-mist, **(C)** temperature-heaters, **(D)** humidity-fan, **(E)** humidity-mist, and **(F)** humidity-heaters.

Each cross-section from the simulated surface view was overlaid onto the graphs of the actual operation in order to visualize any differences (**Figure 11**). In the case of LED, it was only necessary to consider the change in the growth stage, so it was excluded from the variables for this analysis. In each graph, the dots represent actual operational data and the shaded areas represent the simulation. The actual behavior of temperature (**Figures 11A–C**) was not measured at either end of the full range, but humidity (**Figures 11D–F**) was shown to be almost identical to the simulation over the full range. When analyzing only the actual operational data, a clear correlation was observed between the expected and observed humidity values, but the temperature was ambiguous.

Independent of the trend, the fuzzy system was shown to operate as designed when looking at the graph alone. However, when viewed together with the simulation results, other interpretations are possible. The fan and mist were mostly activated within the bounds of the simulated data in terms of temperature and humidity and each showed similar trends. The heater, on the other hand, greatly deviated from the bounds of the simulation, despite its tendency to increase operation as the humidity increased and to decrease as the temperature decreased. This means that the heaters worked inappropriately. In particular, the duty cycles of the heaters should have been much higher below 30 °C and above 45% humidity. As the maximum inner temperature was outside the ideal range, this inappropriate working range might have been better fit for this system.

#### Correlations between input and output variables

**Figure 12:**
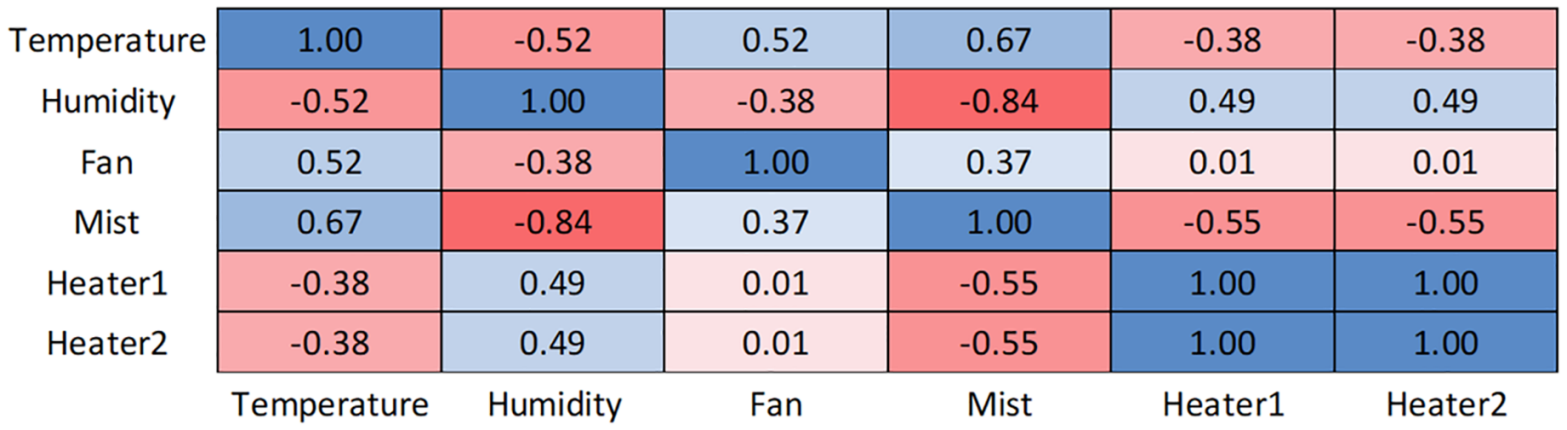
Heatmap of correlations between input and output variables based on Pearson’s correlation coefficients.

The following analysis was performed to examine the cause of the difference between the simulation and the actual operation. A heatmap of Pearson’s correlation coefficients was drawn based on the hypothesis that the input and output variables were closely associated with each other, in order to investigate potential correlations and identify any design flaws in the fuzzy logic (**Figure 12**). This study is ultimately designed to see if the system including LEDs can be controlled as coded. So, correlations were not estimated for LED because each LED should have been operated independently based on the growth stage.

The strongest correlation was between mist and humidity (-0.84), demonstrating a strong negative correlation between the two as the humidifier operated strongly when humidity decreased. Furthermore, mist had a strong positive correlation with temperature and a negative correlation with the heaters (0.67 and -0.55, respectively), that is, when the temperature increased, the humidifier operated to reduce it. Because both mist and heaters were output variables, it could be interpreted that the heaters were on and humidifier was off if the system was heated. In addition, temperature exhibited a strong correlation with humidity and the fan (-0.52 and 0.52, respectively), that is, at lower temperatures, humidity remained high and as the temperature fell, the fan operated less. Considering all of these together, as the temperature increased, the fan and humidifier were operational, while the heaters were switched off in order to cool the cultivator.

The variables that were moderately correlated were: temperature-heaters, humidity-heaters, mist-fan, and humidity-fan. As the temperature increased, the heaters considerably reduced their operations. The mist and fan were coupled because they were both output variables. As humidity increased, the heaters operated to a greater extent, while the fan was moderately active. Lastly, the fan and heaters exhibited no correlation (correlation coefficient = 0.01).

The following problems were identified based on the correlation coefficients. First, the fan should have been strongly correlated with both the mist and heaters, since in addition to the fan, the mist and heater significantly enhanced their temperature and humidity control. However, Pearson’s correlation coefficients indicate that this was not the case (0.37 and 0.01, respectively). This shows that the system entirely missed the correlation between the heaters and the fan. Although the mist and fan exhibited a positive correlation, it was still weak. Ideally, both coefficients should have been > 0.5. To address this, the rules for the fan, mist, and heaters should be redesigned and applied so that humidity and temperature can be effectively controlled. Second, humidity and the fan had a lower correlation than expected, which was presumably why humidity remained > 65%. This could be addressed by modifying the rules and membership functions for humidity and the fan. Finally, the correlation between temperature and the heaters was far weaker than expected. The heaters and mist had a direct effect on the temperature, which should mean strong correlations with temperature. However, the heaters showed a moderate correlation of -0.38 with temperature, while mist showed a strong correlation of 0.57. If the rules and membership functions for temperature and heaters were modified, the desired results could be achieved. Considering these solutions, it is expected that overall performance improvement will be achieved if modifications to the operation of heater and fan are performed.

## Conclusion

We successfully developed a fuzzy logic-controlled system for the home cultivation of sweet basil. Specifically, the light quality was controlled by selecting three growth stages, according to the BBCH scale, as an input variable and assigning three lights (red, blue, and green) as independent output variables. Analysis of the simulation and actual operation data was conducted and most variables, except for the heaters, operated as intended. In order to identify potential design flaws in the fuzzy logic, the correlations between the input and output variables were investigated using scatter plots and Pearson’s correlation coefficients. As a result, the correlations between the heaters and the fan were the weakest. The fuzzy logic control could be improved with appropriate modifications to the operation of heaters and fan. In future studies, the weakest elements of the fuzzy logic and re-implementation of the control system would be necessary for the improved operation of the system. Furthermore, an RGB camera could be used to detect the plant growth stage as another fuzzy logic input parameter instead of time duration. This could allow for a more accurate adjustment of output variables according to environmental conditions as the speed of plant growth can differ among individuals.

## Data availability statement

The raw data supporting the conclusions of this article will be made available by the authors, without undue reservation.

## Author contributions

J-SK designed the study and performed lab experiments. WJ and SP made hardware. J-SK and MY drafted the manuscript. MY reviewed and edited the manuscript. All authors contributed to the article and approved the final manuscript.

## Funding

This work was supported by the National Research Foundation of Korea(NRF) grant funded by the Korea government(MSIT) (No. 2022R1C1C2005959).

## Acknowledgments

This work was supported by the BK21 FOUR, Global Smart Farm Educational Research Center, Seoul National University, Seoul, Korea; and Smart Agriculture Innovation Center of Kyungpook National University, Republic of Korea.

## Conflict of interest

The authors declare that the research was conducted in the absence of any commercial or financial relationships that could be construed as a potential conflict of interest.

## Publisher’s note

All claims expressed in this article are solely those of the authors and do not necessarily represent those of their affiliated organizations, or those of the publisher, the editors and the reviewers. Any product that may be evaluated in this article, or claim that may be made by its manufacturer, is not guaranteed or endorsed by the publisher.
